# Characterization of Thermophilic Halotolerant *Aeribacillus pallidus* TD1 from Tao Dam Hot Spring, Thailand

**DOI:** 10.3390/ijms12085294

**Published:** 2011-08-17

**Authors:** Montri Yasawong, Supatra Areekit, Arda Pakpitchareon, Somchai Santiwatanakul, Kosum Chansiri

**Affiliations:** 1 Department of Biochemistry, Faculty of Medicine, Srinakharinwirot University, Bangkok 10110, Thailand; E-Mails: vimandin@hotmail.com (M.Y.); jeedkha@hotmail.com (S.A.); oom_arda@hotmail.com (A.P.); 2 Department of Pathology, Faculty of Medicine, Srinakharinwirot University, Bangkok 10110, Thailand; E-Mail: titi41@yahoo.com

**Keywords:** thermophile, halotolerant, *Aeribacillus*, pectate lyase

## Abstract

The bacterial strain TD1 was isolated from Tao Dam hot spring in Thailand. Strain TD1 was Gram positive, rod-shaped, aerobic, motile, and endospore forming. The cell was 2.0–40 μm in length and about 0.4 μm in diameter. The optimum growth occurred at 55–60 °C and at pH 7–8. Strain TD1 was able to grow on medium containing up to 10% NaCl. The DNA G+C content was 38.9 mol%. The cellular fatty acid content was mainly C_16:0_, which comprised 25.04% of the total amount of cellular fatty acid. 16S rDNA showed 99% identity to *Aeribacillus pallidus* DSM 3670^T^. Bayesian tree analysis strongly supported the idea that strain TD1 is affiliated with genus *Aeribacillus*, as *Aeribacillus pallidus* strain TD1. Although the 16S rDNA of *A. pallidus* strain TD1 is similar to that of *A. pallidus* DSM 3670^T^, some physiological properties and the cellular fatty acid profiles differ significantly. *A. pallidus* strain TD1 can produce extracellular pectate lyase, which has not been reported elsewhere for other bacterial strains in the genus *Aeribacillus. A. pallidus* strain TD1 may be a good candidate as a pectate lyase producer, which may have useful industrial applications.

## 1. Introduction

Thermophiles, especially thermophilic enzymes, have attracted much interest as both analytical tools and biocatalysts for applications at a large scale [1]. The application of robust enzymes and microorganisms for the sustainable production of chemicals, biopolymers, materials, and fuels from renewable resources, defined as industrial biotechnology, offers many opportunities for various industries. The most important involve applications to the reduction of waste, energy input, and raw materials, and the development of highly efficient and environmentally friendly processes [2]. Pectate lyase (EC 4.2.2.2), or pectate transeliminases, catalyzes the eliminative cleavage of de-esterified pectin, a major component of the primary cell walls of many higher plants [3]. Potential uses of pectinase include extraction and clarification of fruit juices and wine, maceration of vegetables, scouring of cotton fabric, retting of flax, degumming of plant fibers, fermentation of coffee and tea, as poultry feed, in oil extraction, and pretreating wastewater containing pectinacious material [4]. Pectinases have been isolated from various microbial sources such as bacteria [5–8], yeast [9] and fungi [10,11]. Recently, thermostable pectate lyases were isolated and characterized from many mesophilic and thermophilc bacteria such as *Bacillus* sp. RN1 [12–14]. The strain was isolated from a hot spring in Thailand and produced pectate lyase (Pel SWU), which can be applied in many industrial fields [13].

Hot springs are a rich source of thermophiles. More than 100 hot springs are distributed throughout Thailand, and the surface temperatures are in the range 40–100 °C at a pH range of 6.4–9.5. Tao Dam hot spring was discovered in 2008. The spring is located in the central part of Thailand. Water in this spring contains a high concentration of sulfide. Surprisingly, this sulfide-rich spring has a pH greater than seven, which is rare in sulfide-rich hot springs in Thailand.

Given the important role of thermophiles in biotechnology, the present study attempted to characterize thermophilic bacteria from Tao Dam hot spring, which contain pectate lyase activity. The study used bacterial culture, microscopic observations, and phenotypic, chemotaxonomic, phylogenic, and molecular systematic analysis to characterize these bacteria.

## 2. Results and Discussion

**Figure 1.**
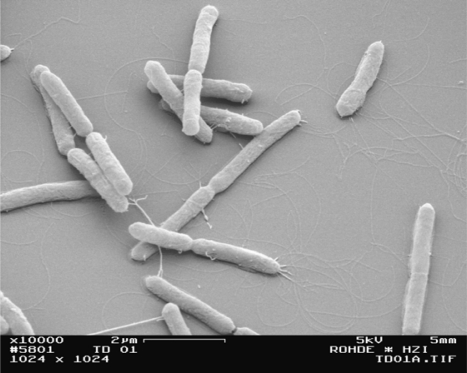
Scanning electron micrograph of strain TD1. Bar, 2 μm.

Fourteen bacterial strains were isolated from the spring sediment. Only strain TD1 was able to produce clear halo zones on agar medium containing 1% AZ rhamnogalacturonan. A photometric assay indicated that extracellular pectate lyase was produced by TD1. The morphology of strain TD1 was rod-shaped and contained lophotrichous flagella (Figure 1).

**Figure 2.**
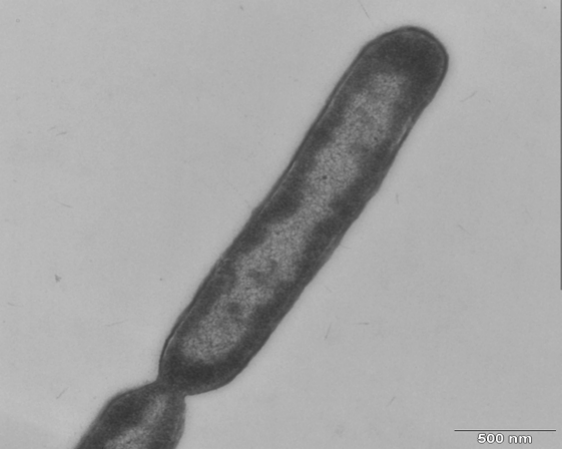
Electron micrograph of ultrathin-sectioned of strain TD1.

Gram staining and type were positive. The cell was motile. Each cell was 2.0–40 μm in length and about 0.4 μm in diameter (Figure 2). The endospore was ellipsoidal and was located in the subterminal swollen sporangium. The temperature range for growth was 30–67 °C, and the optimum growth conditions were 55–60 °C and pH 7–8. Strain TD1 was able to grow in medium containing 0–10% NaCl. The physiological characteristics of the strain TD1 are shown in Table 1.

The utilization of carbohydrates differed markedly between strain TD1 and the *A. pallidus* type strain (Table 1). Only strain TD1 was able to produce acid from arabinose, cellobiose, mannose, ribose, and xylose. Strain TD1 could utilize citrate and propionate. Hydrolysis of starch was not detected in strain TD1.

The cellular fatty acid profile of strain TD1 is shown in Table 2. The cellular fatty acid was mainly C_16:0_, which comprised 25.04% of the total amount of cellular fatty acid. The cellular fatty acid profiles differed significantly between strain TD1 and *A. pallidus* DSM 3670^T^. *A. pallidus* DSM 3670^T^ contained more than twice the amount of C_16:0_ compared with strain TD1 (Table 2).

**Figure 3.**
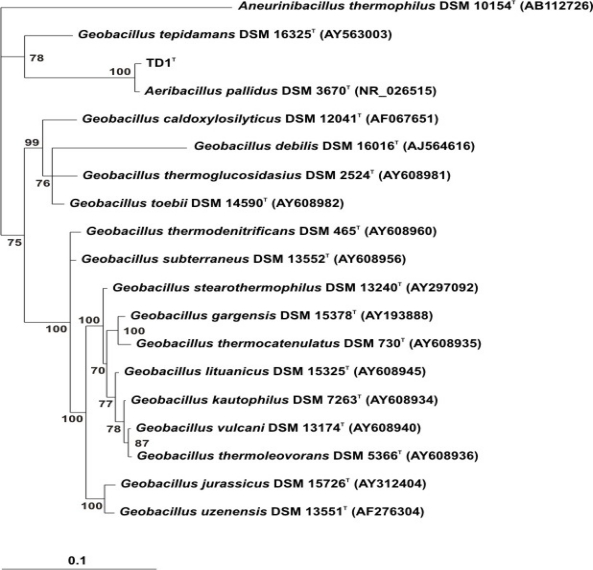
The Bayesian tree was constructed using the Bayesian inference algorithm with the GTR+I+G model of nucleotide substitution. The values associated with nodes correspond to the clade credibility support in %.

Strain TD1 contained linear and branched fatty acids, and some unsaturated fatty acids; branched saturated fatty acids were dominant. By contrast, no unsaturated fatty acids were detected in strain *A. pallidus* DSM 3670 and linear fatty acids were dominant. The G+C content was 38.9 mol%. The 16S rDNA of strain TD1 (GQ355275) was similar to that of *A. pallidus* DSM 3670^T^ (99% identity). The Bayesian tree is shown in Figure 3. Strain TD1 clustered with *A. pallidus* DSM 3670^T^ (100% clade credibility).

The Bayesian tree strongly supports the idea that strain TD1 is affiliated with genus *Aeribacillus* as *Aeribacillus pallidus* strain TD1. At the time of writing, *A. pallidus* DSM 3670^T^ is the type and sole species of genus *Aeribacillus* [16]. This strain was first described in 1987 by Scholz and colleagues as “*Bacillus pallidus*” [15] and was renamed in 2004 by Banat and colleagues as “*Geobacillus pallidus*” [17]. Based on 16S rDNA sequence divergence and the presence of unique phenotypic characteristics, the strain was then transferred to the new genus *Aeribacillus* as *A. pallidus* in 2010 by Minana-Galbis and colleagues [18].

## 3. Experimental Section

### 3.1. Isolation of Bacterial Strains

Bacterial strains were isolated from Tao Dam hot spring, Khluang Wangchao National Park, Thailand, which is located in a remote region of Kampangpetch province. The spring was discovered recently and contains mineral (sulfide-rich) water with a surface temperature range of 50–75 °C and pH 8.0. The sediment was 60 °C at the time of sampling. The bacteria were isolated using tenfold serial dilutions of sediment in phosphate buffer (0.1 M K_2_HPO_4_ and 0.1 M KH_2_PO_4_, pH 8). The dilutions were spread on R2A agar (Difco, NJ, USA), and the plates were incubated overnight at 55 °C.

### 3.2. Pectate Lyase Assay

Bacterial isolates and the type strain of *A. pallidus* DSM 3670 were grown on R2A agar which contained one percent of AZ rhamnogalacturonan (Megazyme, Ireland). The plates were incubated overnight at 55 °C. A clear halo zone should appear when pectate lyase is produced. Positive clones were inoculated in 100 mL of M9 broth (pH 8) plus 1 g sodium pectate (Sigma, Germany). The flask was incubated at 55 °C with shaking at 150 rpm overnight. Bacterial cells were removed by centrifugation at 8,000 rpm for 10 min, and the supernatant was collected for the photometric assay, in which 0.5 mL of supernatant was added to 2.5 mL of substrate (60 mM Tris-HCl, pH 8.0, 0.6 mM CaCl_2_, and 0.24% (w/v) sodium pectate). The reaction was incubated at 55 °C for 2 h. Pectate lyase activity was measured on a Genesys 10 uv spectrophotometer (Thermo Fisher Scientific, Madison, WI, USA) at 232 nm as described by Collmer and colleagues [19]. Pectate lyase from *Aspergillus* sp. (Megazyme) was used as the positive control, and sterile water and substrate were used as the negative control.

### 3.3. Morphology

The morphology was examined under a Zeiss Gemini DSM852 field emission scanning electron microscope (Zeiss, Oberkochen, Germany) and Zeiss transmission electron microscope TEM910 (ProScan, 1024 × 1024, Scheuring, Germany). To prepare the samples for field emission scanning electron microscopy, the samples were fixed with a solution containing 2% glutaraldehyde and 5% formaldehyde in cacodylate buffer (0.1 M cacodylate, 0.09 M sucrose, 0.01 M MgCl_2_, and 0.01 M CaCl_2_, pH 6.9) for 1 h on ice and then washed with TE buffer (20 mM Tris and 2 mM EDTA, pH 6.9). The fixed bacterial cells were placed onto poly-l-lysine-coated cover slips, left for 5 min, washed once in TE buffer, and fixed with 2% glutaraldehyde. After washing with TE buffer, the samples were dehydrated with a graded series of acetone (10%, 30%, 50%, 70%, 90%, 100%) for 10 min at each step on ice, except that the final 100% acetone step was performed at room temperature. Samples were critical-point dried with liquid CO_2_ (CPD 030, Bal-Tec, Liechtenstein), mounted onto aluminum stubs, sputter coated with gold (SCD 500, Bal-Tec), and examined in the field emission scanning electron microscope at an acceleration voltage of 5 kV using an Everhart–Thornley SE-detector and in-lens SE-detector at a 50:50 ratio. Images were stored onto a 230 MB MO-disk.

To embed the bacteria for transmission electron microscopy, the samples were fixed in a fixation solution containing 5% formaldehyde and 2% glutaraldehyde in cacodylate buffer (0.1 M cacodylate, 0.01 M CaCl_2_, 0.01 M MgCl_2_, 0.09 M sucrose, pH 6.9), washed with cacodylate buffer, fixed with 1% aqueous osmium for 1 h at room temperature, and washed. The pellets were embedded in 2% water agar and cut into small cubes. The samples were dehydrated in a graded series of acetone (10%, 20%, 50%) for 30 min on ice followed by contrast staining with 2% uranyl acetate in 70% acetone overnight at 4 °C, and the samples were dehydrated further with 90% and 100% acetone. At the 100% acetone step, the samples were allowed to reach room temperature and were then infiltrated with the epoxy resin according to Spurr’s formula for a medium resin [20]: 1 part 100% acetone/1 part resin overnight; 1 part 100% acetone/2 parts resin for 8 h; and pure resin overnight and several changes in the following 2 d. The samples were then transferred to resin-filled gelatin capsules and polymerized for 8 h at 75 °C. Ultrathin sections were cut with a diamond knife, picked up onto Formvar-coated copper grids (300 mesh), and counterstained with 2% aqueous uranyl acetate and lead citrate. After air-drying, the samples were examined in a Zeiss TEM910 transmission electron microscope at an acceleration voltage of 80 kV. The images were recorded digitally with a Slow-Scan CCD-Camera (ProScan, 1024 × 1024, Scheuring, Germany) with ITEM-Software (Olympus Soft Imaging Solutions, Germany).

### 3.4. Phenotypic Characterization

The temperatures for growth of the strain TD1 were examined from 30–70 °C on R2A medium (Difco, NJ, USA). The salt tolerance was also assessed on R2A medium with 0–10% (w/v) NaCl at 55 °C. The phenotype of the strain TD1 was investigated using API 20E and API 50 CHB tests (bioMérieux, Nürtingen, Germany) at 55 °C, according to the manufacturer’s instructions.

### 3.5. Chemotaxonomy

Analysis of cellular fatty acids was performed at the German Collection of Microorganisms and Cell Cultures (DSMZ) (Braunschweig, Germany) according to the Sherlock Microbial Identification System manual (version 4.5; Microbial ID, Newark, DE). The profiles of cellular fatty acids were compared using the TSBA40 library database version 4.10 (Microbial ID, Newark, DE, USA).

### 3.6. Molecular Systematics

DNA was extracted using MasterPure™ Gram Positive DNA Purification Kit (Biozym Scientific, Germany) according to the manufacturer’s instructions. The genomic G+C ratio was examined using HPLC at DSMZ. Primers 27f (5′-AGAGTTTGATCMTGGCTCAG-3′) and 1492r (5′-TACGGYTACCTTGTTACGACTT-3′) [21] were used for bacterial 16S rDNA amplification. The 50 μL total volume of PCR reaction contained 10 ng of DNA, 20 pmol of each primer, 80 μM dNTP, 1 × *Taq* reaction buffer, and 1.25 U *Taq* DNA polymerase (EURx, Poland). The PCR was started by heating the reactions to 94 °C for 3 min, followed by 30 cycles of 30 s at 94 °C, 30 sec at 52 °C, and 1 min at 72 °C, and ending with a final step at 72 °C for 3 min. The PCR product was purified using a MinElute PCR Purification Kit (QIAGEN, Germany). DNA sequencing was performed directly on the purified PCR product. The sequencing primers were 27f and 1492r. Big Dye v. 1.1 reagents (Applied Biosystems, Inc.) were used for the sequencing, and the analysis was performed on an ABI Genetic Analyzer sequencer (AB3130XL). The sequence was compared with the nonredundant database of sequences deposited at GenBank using BLAST [22]. 16S rDNA of the strain TD1 was aligned with other bacterial strains in the genera *Geobacillus* and *Aeribacillus* using MUSCLE 3.7 [23]. The size of the 16S rDNA for the alignment was about 1,500 bp. The evolutionary model was calculated using Modeltest 3.8 [24]. Bayesian inference was performed using MrBayes 3.1.2 [25]. The Bayesian posterior probabilities were obtained by performing two separate runs with four Markov chains. Each run was conducted with 2 × 10^6^ generations and sampled every 100 generations. Convergence was checked by examining the generation plot visualized with TRACER 1.4 [26]. Potential scale reduction factor was computed by MrBayes 3.1.2. A consensus tree was calculated after discarding the first 25% of the iterations as burn-in.

## 4. Conclusions

*A. pallidus* strain TD1 is a thermophilic and halotolerant bacterium. The physiological properties of strain TD1 are promising for biotechnology research. *A. pallidus* strain TD1 can produce extracellular pectate lyase, which has not been reported for other bacterial strains in the genus *Aeribacillus. A. pallidus* strain TD1 may be a good candidate as a pectate lyase producer and may have useful industrial applications.

## Figures and Tables

**Table 1.** Physiological characteristics of *A. pallidus* strain TD1 and *A. pallidus* DSM 3670^T^ [15].

**Characteristic**	***A. pallidus* TD1**	***A. pallidus* DSM 3670^T^**
Cell width (μm)	0.4	0.8–0.9
Cell length (μm)	2–>40	2–5
Motility	+	+
Temperature range (°C)	45–67	30–70
DNA G+C content (mol%)	38.9	39–41
Producing of pectate lyase	+	−
Acid produced from:		
Cellobiose	+	−
Maltose	+	d
Mannose	+	−
Sucrose	+	ND
Trehalose	+	d
Xylose	+	−
Arabinose	−	−
Ribose	+	−
Citrated used	+	−
Hydrolysis of:		
Casein	−	−
Gelatin	−	−
Strach	−	+^w^
Alkane utilization	−	ND

+, positive;

−, negative;

+^w^, weakly positive;

d, variable between strains;

ND = not determined.

**Table 2.** Cellular fatty acid profiles of *A. pallidus* TD1 and *A. pallidus* DSM 3670^T^ [15].

**Fatty Acid**	***A. pallidus* TD1 (%)**	***A. pallidus* DSM 3670^T^ (%)**
14:0 ISO	0.36	1.6
14:0 ANTEISO	–	1.6
14:0	1.87	8.5
15:0 ISO	16.30	6.2
15:0 ANTEISO	4.50	4.9
15:0	0.33	1.2
16:1 w7c alcohol	0.14	–
16:0 ISO	11.15	9.3
16:1 w11c	0.65	–
16:0	25.04	50.0
ISO 17:1 w10c	0.30	–
17:0 ISO	17.95	4.0
17:0 ANTEISO	19.74	6.5
17:0	0.19	–
18:0 ISO	0.29	–
18:0	1.03	2.1
